# Magnetic resonance velocity imaging derived pressure differential using control volume analysis

**DOI:** 10.1186/2045-8118-8-16

**Published:** 2011-03-17

**Authors:** Benjamin Cohen, Abram Voorhees, Timothy Wei

**Affiliations:** 1 Mechanical, Aerospace and Nuclear Engineering, Rensselaer Polytechnic Institute, 110 8th Street, Troy, NY 12180, USA; 2 Siemens Healthcare, 51 Valley Stream Parkway, Malvern, PA 19355, USA

## Abstract

**Background:**

Diagnosis and treatment of hydrocephalus is hindered by a lack of systemic understanding of the interrelationships between pressures and flow of cerebrospinal fluid in the brain. Control volume analysis provides a fluid physics approach to quantify and relate pressure and flow information. The objective of this study was to use control volume analysis and magnetic resonance velocity imaging to non-invasively estimate pressure differentials *in vitro*.

**Method:**

A flow phantom was constructed and water was the experimental fluid. The phantom was connected to a high-resolution differential pressure sensor and a computer controlled pump producing sinusoidal flow. Magnetic resonance velocity measurements were taken and subsequently analyzed to derive pressure differential waveforms using momentum conservation principles. Independent sensor measurements were obtained for comparison.

**Results:**

Using magnetic resonance data the momentum balance in the phantom was computed. The measured differential pressure force had amplitude of 14.4 *dynes *(pressure gradient amplitude 0.30 *Pa/cm*). A 12.5% normalized root mean square deviation between derived and directly measured pressure differential was obtained. These experiments demonstrate one example of the potential utility of control volume analysis and the concepts involved in its application.

**Conclusions:**

This study validates a non-invasive measurement technique for relating velocity measurements to pressure differential. These methods may be applied to clinical measurements to estimate pressure differentials *in vivo *which could not be obtained with current clinical sensors.

## Background

Hydrocephalus is a complex spectrum of neuropathophysiological disorders generally defined by increased cerebrospinal fluid (CSF) within the cerebral ventricles and elevated intracranial pressure (ICP) [1]. The diagnosis and treatment of hydrocephalus is hindered by a lack of systemic understanding of the interrelationships between pressures and flow of CSF in the brain. Advancements in interpreting and combining clinical measurements within a precise, direct, physics-based approach will improve quantification, understanding and diagnosis of hydrocephalus. Control volume analysis was proposed to incorporate clinical measurements within a simple, robust fluid dynamics analysis [2]. This method allows direct quantitative comparison between disparate data sets through the integral mass and momentum conservation equations, precluding the need for analogies between physics and physiological significance. Inherently the fundamental nature of control volume analysis allows great flexibility when applying such an approach [2]. One potentially useful application of control volume analysis is the estimation of *in vivo *pressure differentials from non-invasive velocity images. This approach represents an advancement in interrelating flow and pressure information *in vivo *through a precise, physically meaningful framework.

Ultimately, control volume formulations of CSF and blood flow in the brain may be performed as a scientifically rigorous means of quantifying pressure variations throughout the brain. However, the purpose of this study was to use control volume analysis and phase-contrast magnetic resonance (PC-MR) imaging to non-invasively estimate pressure differentials within a flow phantom as a proof-of-concept prior to *in vivo *studies.

### *In vivo *CSF pressure measurements

Pressure differential is the force driving fluid flow in the CSF system [3,4]. Measurement of *in vivo *pressure differential currently poses several technical challenges. Generally, intracranial sites may be inaccessible or implausible for implanted sensors and hindered by invasive implantation into soft biological tissue. In addition, measurements are complicated by the meager pressure differentials expected between freely communicating fluid spaces (e.g. transmantle pressure). Therefore, determining the pressure difference by measuring (gage) pressure at two locations inherently introduces significant errors; related to inadequate sensor resolution and accuracy, calibration and drift, and changes in head position over time [5,6].

Conner *et al*. found the transmantle pressure to be 0.27 ± 0.31 *cm saline *(~27 ± 31 *Pa*) prior to kaolin induction of hydrocephalus in cats, which then increased to 3.4 ± 3.9 *cm saline *post induction [7]. Penn *et al*. noted the lack of a consistent pressure difference between the lateral ventricle, frontal lobe, and anterior subarachnoid space in dogs using the InSite monitor system (Medtronic Neurological), with a pressure resolution of 0.5 *mmHg *(66 *Pa*). The authors concluded that if pressure gradients existed, they were smaller than the sensor resolution [5]. Rekate *et al*. obtained a differential pressure resolution of 0.15 *mmHg *(20 *Pa*) in greyhounds using manometric techniques. Pressure differences were only observed during external withdrawal of CSF [8].

Experiments in human subjects have provided mixed results. In three control subjects a transmantle pressure of ~ 70 ± 120 *Pa *was found compared to ~ 480 ± 430 *Pa *in eleven patients with normal pressure hydrocephalus [7]. Stephenson *et al*. performed carefully monitored experiments and reported no transmantle pressure difference existed, -1.33 ± 32 *Pa*, within experimental uncertainty [6].

These experimental measurements suggest that physiologically relevant (time-averaged) ICP differentials, if they exist, are less than ~ 30 *Pa*. Numerical simulation results support this assertion. Two-dimensional Navier-Stokes simulations of the intracranial space predicted transmantle pressure remains below 10 *Pa*, while the ICP pulse amplitude was ~ 27 *Pa *[9]. Computational studies of CSF flow in the cerebral aqueduct required a pressure drop of ~ 1 *Pa *over the 1.1 *cm *length of the domain to sustain bulk flow rates observed *in vivo *(0.36 *mL*/*min*) [4].

### Phase-contrast magnetic resonance velocity measurements

Velocities of the intracranial contents have been measured throughout the cardiac cycle using velocity encoded cine phase-contrast magnetic resonance (PC-MR) imaging. PC-MR produces images in which pixel intensity is proportional to velocity. *In vivo *blood [3,10-14] and CSF velocities [3,10,11,15-17] have been measured using this technique. PC-MR is capable of measuring up to three velocity components volumetrically, however to improve spatial and temporal resolution and decrease scan time typically through-plane velocities are measured within a single slice perpendicular to the flow direction [18]. Through-plane PC-MR images are sensitized to velocity values between ±*V_enc_*, the encoding velocity. A number of investigators have used through-plane velocity data to compute volume flow rate waveforms *in vivo *[3,10-12,15,17]. Frayne *et al*. obtained through-plane velocity measurements in a pulsatile flow phantom and found the root mean square (rms) difference to be 1.6 *cm*/*s*, 7.5% of the mean velocity [19]. Wentland *et al*. made similar measurements for velocities typical of those observed in foramen magnum CSF flow (1-20 *cm*/*s*) and obtained larger errors at low velocities [17].

### Non-invasive pressure estimation *in vivo*

Several novel approaches can be found in the literature describing methods to non-invasively estimate pressure *in vivo*. Ohara *et al*. found statistically significant correlation between signal-void phenomenon and ICP, implying that ventricular CSF signal intensity in MR images can potentially be used to differentiate between normal and raised ICP [20]. Reid *et al*. described pilot studies to monitor ICP non-invasively by measuring tympanic membrane displacement within the middle ear [21]. In addition, ultrasonography techniques have been used to estimate ICP non-invasively [22,23].

Urchuk and Plewes [24] used the Navier-Stokes equation to estimate pressure gradient waveforms from PC-MR velocity measurements within rubber tubing. The authors applied spatial and temporal difference operators to velocity data to determine the pressure gradient waveform, consisting of inertial and viscous contributions. The purpose of the study was to compare PC-MR derived pressure gradient waveforms to transducer measurements, for pressure gradients on the order of 0.01 - 2.0 *mmHg*/*cm *(1.33 - 267 *Pa*/*cm*). The quoted precision based on these experiments was 0.01 - 0.03 *mmHg*/*cm *(1.33 - 4.0 *Pa*/*cm*), which is the upper magnitude bound for the pressure gradients existing in CSF [3,4].

Alperin *et al*. have devised a method to non-invasively estimate intracranial elastance and ICP from flow-sensitive MR imaging assuming a monoexponential elastance curve [10,25]. The authors performed correlation studies on computers [26] and in baboons to derive relations between time variations in ICP and pressure gradient. Pressure gradient waveforms, estimated based on the method of Urchuk and Plewes [24], were obtained in the cervical spinal canal along with PC-MR volume flow measurements allowing elastance and ICP to be estimated.

## Methods

Due to the variability of physiological systems a flow phantom was used as a proof-of-concept for determining pressure differentials using control volume analysis [2]. Additionally, several difficulties of pressure instrumentation and measurement in animals were overcome with a model system. The flow phantom was designed to mimic the CSF spaces of the cranium and a pump conducts time varying volume flow into the phantom, representing those seen *in vivo *at the cranio-cervical junction.

### Theory

Conservation laws are commonly used in physics to relate important variables of interest. Hydrocephalus research and modeling has been no exception. In control volume analysis, conservation principles are enforced within finite volumes, termed control volumes (CV). Control volumes are clearly defined regions which are used in conjunction with the integral conservation equations to analyze fluid flow. For any CV the mass and momentum conservation equations must be satisfied. Stated simply, these equations enforce the observations that mass cannot be created or destroyed and that the time-rate of change of momentum of a specified mass is equal to the net force applied. Momentum conservation in fluids is equivalent to Newton's second law for solid objects. The mathematical formulation is presented here for completeness, with mathematical symbols defined in Table 1. Cohen *et al*. provided a detailed physical description of the control volume formulation [2].

**Table 1:** Physical quantities, corresponding symbols and MKS and CGS units

Physical Quantity	Symbol	MKS units	CGS units
Fluid mass density	*ρ*	*Kg*/*m*^3^	*g*/*cm*^3^
Velocity vector field	**U**	*m*/*s*	*cm*/*s*
Net mass production rate		*kg*/*s*	*g*/*s*
Pressure field	*p*	*Pa*	*dyne*/*cm*^2^
Viscous stress vector	***τ***	*Pa*	*dyne*/*cm*^2^
Gravitational acceleration	**g**	*m*/*s*^2^	*cm*/*s*^2^
Control volume acceleration	**a**	*m*/*s*^2^	*cm*/*s*^2^
Ventricle wall displacement vector	***δ***_**w**_	*m*	*cm*
Differential volume element	*dV*	*m*^3^	*cm*^3^
Differential control surface area element	*dS*	*m*^2^	*cm*^2^
Unit outward surface normal vector		--	--

The mass conservation equation for a CV states that the time-rate of change of mass within the CV must equal the net mass flow into the CV through the control surface (CS) plus the net production (production minus absorption) of mass within the CV. This last term is included to account for naturally occurring production and absorption of CSF. This simplifies analysis because only velocities at the CS and net changes within the CV enter in the formulation.

The conservation of mass equation for a general CV is written:(1)

where:

(*i*) Rate of change of fluid mass in the control volume

(*ii*) Net mass flow rate across the control surface

(*iii*) Net mass production/absorption rate within the control volume

Momentum conservation is a generalized statement of Newtons's second law; the resultant force acting on a mass is balanced by the time-rate of change of momentum of the specified mass. Similarly, the time-rate of change of the momentum inside the CV must equal the forces acting on the CV, including those created by fluid flow crossing the CS. The conservation of momentum equations for a general CV are written:(2)

where:

(*i*) Rate of change of fluid momentum in the control volume; inertial force

(*ii*) Net fluid momentum flow rate across the control surface; force due to fluid flow

(*iii*) Body force due to gravity and acceleration of mass in the control volume

(*iv*) Pressure force acting on the control surface

(*v*) Viscous force acting on the control surface

In these equations the symbols represent physical quantities presented in Table 1. Bold type is used to represent vector quantities.

### Experimental Study

The purpose of the flow phantom was to study a repeatable system in which the pressure differential derived through the conservation equations could be independently measured using a differential pressure sensor of adequate resolution.

**Figure 1:**
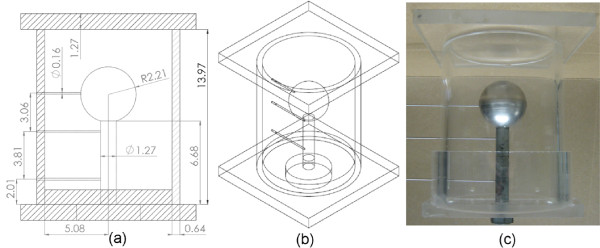
**Computer drafted model and photo of flow phantom**. (a) Dimensioned drawing of the experimental flow phantom. Hatching denotes acrylic parts in the actual phantom. Dimensions in *cm*. (b) Isometric view of flow phantom model. (c) Photo of phantom, in similar orientation to (a), taken prior to agarose gel being poured around metal. Once agar is cured, the metal is removed, creating voids in the gel for water. Three small holes are reserved for pressure taps.

Figure 1 shows computer aided drafts and a photo of the flow phantom; important dimensions are shown in Figure 1(a) and in isometric view in Figure 1(b). Phantom fabrication followed the lost-material casting technique presented by Smith *et al*. using low melting point metal (Cerrolow 117, Cerro Metal Products, Bellefonte, PA, USA) [27]. The finished phantom consisted of an acrylic container, shown by hatching in Figure 1(a), partially filled with agarose gel (AG-LE, MB Grade Agarose, Lab Scientific, Livingston, NJ, USA; 2% weight/volume water). The gel contained a water filled spherical cavity, representing the cerebral ventricular system, communicating with a cylindrical passage as shown in Figure 1. Figure 1(c) shows the phantom prior to the agarose gel being poured and the metal removed, leaving cavities to be filled by water. Water was used as the working fluid to represent CSF. Plastic connectors were integrated into the base of the phantom allowing it to be connected to (and disconnected from) tubing. The phantom was instrumented with three pressure taps as shown in Figure 1. Only two were connected to the differential pressure sensor during pressure measurements.

The phantom was designed to match the Reynolds number, , and Womersley number, , found at the cervical apex of the spinal canal as reported by Loth *et al. *[26]. Here Ū is the mean velocity at the time of peak flow, *D_h _*is the hydraulic diameter, *ν *is the dynamic viscosity of the fluid and *ω *is the angular frequency. For convenience, the Reynolds number was defined by the maximum flow rate in the phantom, , as  where *D *= 1.27 *cm *is the diameter of the cylindrical passage in the phantom as shown in Figure 1(a). The hydraulic diameter was used to relate the quasi-annular geometry of the spinal canal to the cylindrical passage in the phantom. Matching dimensionless fluid dynamic parameters, *Re *and *α*, provided realistic values for quantities of interest in this study.

**Figure 2:**
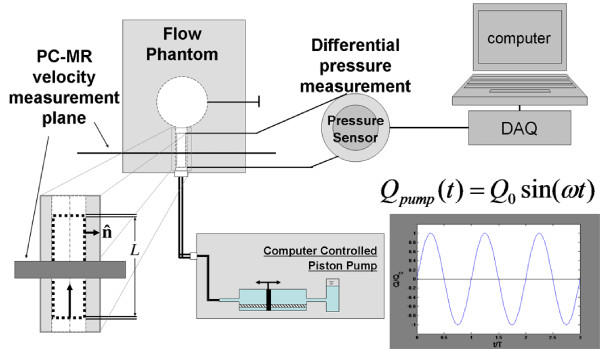
**Schematic of the flow phantom experiments**. During experiments the pressure taps were in a horizontal plane (ie. gravity is perpendicular to the figure plane). The cylindrical passage is enlarged to show the control volume of interest with axial length *L *between pressure taps. Long bold arrow points in axial direction and short bold arrow depicts the unit outward surface normal vector, . PC-MR velocity measurements were obtained independent of pressure data under the same imposed flow waveform. DAQ - Data acquisition electronics.

Figure 2 illustrates the experimental set up of the flow phantom and the desired measurements. A computer controlled piston pump (CompuFlow 1000, Shelley Medical Imaging, Toronto, Ontario, Canada) supplied sinusoidal, zero-net volume flow rate waveforms, *Q_pump_*(*t*) = *Q*_0_*sin*(*ωt*), into and out of the phantom at its base, where  and the volume flow rate amplitude was controlled using the pump. A frequency of one Hertz was chosen to approximate the human heart rate; the principle driver of transcranial CSF pulsations. The phantom was connected to the pump through ~ 6 *m *of braid-reinforced, high-pressure, 0.635 *cm *inner diameter tubing allowing the pump to be outside the MR imaging scanner room. A three-way, two-position valve was used to divert fluid back into the pump during the initial piston transient (not shown in Figure 2).

Pressure tubing, 0.8 *mm *inner diameter spaghetti tubing, connected each side of the differential pressure sensor (LPM9481, GE Sensing/Druck, Houston, TX, USA) to a tap in the phantom communicating with the internal fluid cavities, as shown in Figure 2. During experimentation the phantom was oriented such that the pressure taps were on the horizontal mid-plane and unused pressure taps were purged of air and plugged. Data acquisition electronics (NIcDAQ-9172 and NI9201, National Instruments, Austin, TX, USA) and a personal computer running LabVIEW were used to record the pressure sensor and piston pump output at 5 *kHz*. All pressure measurements were made in lab at Rensselaer while MR measurements were made in a MR facility with the same experimental setup and parameters.

### PC-MR Acquisition

MR examination of the flow phantom was carried out using a standard quadrature birdcage head coil in a 3T MR imaging scanner (Magnetom TrioTim, Siemens, Erlangen, Germany). The phantom was placed at the center of the head coil and stabilized with foam blocks. A retrospectively gated, through-plane PC-MR sequence was used with the following parameters: flip angle = 30°, number of signal averages = 2 - 5, TR = 52 - 76 *ms*, TE = 4.6 - 8.2 *ms*, *V_enc _*= 5 - 20 *cm*/*s*, 11 - 17 cardiac phases, 10 *mm *slice thickness, 128*x*128 matrix, 25.6 *cm *field of view and flow encoding in the right/left direction. A pulse generator was used to simulate the cardiac gating signal at 60 beats per minute. The approximate location of the PC-MR velocity measurement plane in relation to the phantom is shown in Figure 2.

### PC-MR post-processing

PC-MR imaging provides velocity data which may be interpreted through the mass and momentum conservation equations [2]. Direct computation of terms in Equations (1) and (2), based on their physical meaning, provides estimates of physical quantities of interest, such as volume flow rate and pressure differential, and a scientifically rigorous method to interrelate them. This section introduces new methods to analyze PC-MR velocity data based directly on CV principles.

Custom software implemented in MATLAB (R2007a, ver. 7.4, The MathWorks, Inc., Natick, MA, USA) was used to determine the region of interest and estimate terms in the conservation equations. For the interested reader, more details and a mathematical description of these methods can be found in Additional File 1: PC-MR post-processing methodology.

**Figure 3:**
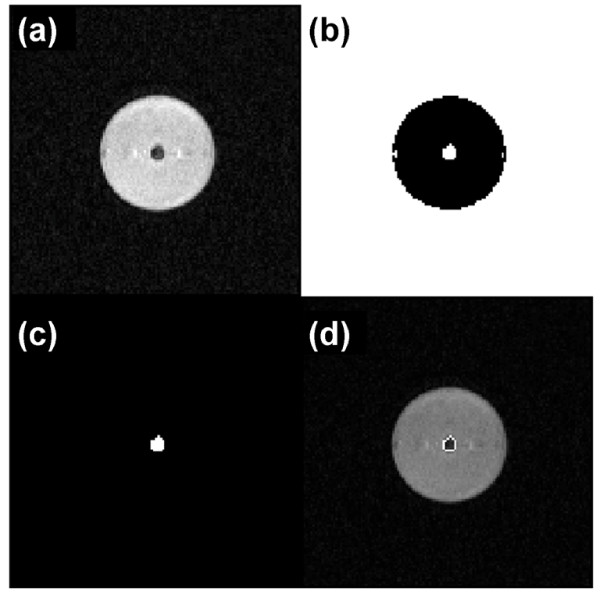
**Region of interest segmentation**. (a) Process of static ROI segmentation begins with magnitude image. (b) User defined threshold is applied creating a black and white image. (c) User manually selects desired bright region in (b) leaving the ROI of interest. (d) The perimeter pixels of the ROI are combined with original magnitude image to ensure ROI determination accuracy.

A region of interest (ROI) was segmented from the PC-MR data sets. Figure 3 outlines the process of ROI segmentation. Ideally the ROI contains only pixels within the lumen of the vessel of interest and represents the portion of the control surface through which fluid flows. Terms in the conservation equations are estimated from velocity values within the ROI.

The volume flow rate, *Q*(*t*), is the volume of fluid passing through a control surface per unit time and is related to mass flow rate, term (*ii*) in Equation (1), through the fluid density, *ρ*. As mentioned previously, numerous investigators have calculated volume flow rate waveforms *in vivo *using PC-MR measurements [3,10-12,15,17]. However, to our knowledge, no studies have utilized the same velocity data to compute a fluid's momentum flow, inertial, and viscous force terms using integral momentum conservation, Equation (2) [2].

Terms in the axial momentum equation were derived from through-plane PC-MR data normally used for volume flow computations. Momentum flow, term (*ii*) in Equation (2), represents the force flowing fluid imparts on a control volume as it crosses the control surface (e.g. fluid exiting a hose requires restraint to keep it from whipping about). Computation of the axial momentum flow waveform is similar to volume flow; the same ROI is used, however the velocity values at each pixel are squared and multiplied by the fluid density. For pulsatile flow of an incompressible fluid in conduit control volumes, the time-rate of change of momentum, term (*i*) in Equation (2), is primarily due to temporal variations of the fluid velocity field within the CV. When flowing fluid changes velocity (speed and/or direction) it creates an inertial reaction force (e.g. water hammer) on the control volume. Therefore, the axial inertial force is proportional to the temporal derivative of the volume flow rate waveform. Finally, the viscous shear force, term (*v*) in Equation (2), represents drag on the control surface due to fluid friction. To compute the axial shear force on the CV the shear stress at the wall must be integrated over the control surface. The wall shear stress is proportional to the fluid viscosity, *μ*, and the velocity gradient (change in velocity over distance) directed away from the solid surface. These methods were implemented in MATLAB using the mathematical formulation found in Additional File 1: PC-MR post-processing methodology.

Estimates obtained from PC-MR data naturally contain errors, introduced by spatial and temporal discretization of the measured velocity field, relative to the integral expressions of Equations (1)-(2). The method of Urchuk and Plewes [24], for estimating pressure gradient waveforms from PC-MR measurements, was used to compare to control volume estimates. For direct comparison with CV waveforms the pressure gradient waveform, and inertial and viscous contributions, were multiplied by *SL*, the volume of the CV.

The relation for velocity noise standard deviation in two-point velocity images, , was used to determine noise in PC-MR measurements [18,24]. Signal to noise ratio (SNR) was computed as the ratio of signal to noise rms amplitude squared. Error was quantified by using the rms deviation between two waveforms normalized by the range of the expected waveform (i.e. measured or Urchuk/Plewes).

## Results

The CV of interest was selected from the volume occupied by water within the phantom, shown outlined by dashed black lines in Figure 2. The mass and momentum conservation equations, (1) and (2), must hold at all times for this space and all subspaces therein. The cylindrical region of the phantom, outlined by a black dotted line and enlarged in Figure 2 is of particular interest in this manuscript. For this CV the axial length between the pressure taps was *L *= 3.81 *cm*, as shown in Figure 1(a). PC-MR imaging provided axial, through-plane velocities to estimate terms in the conservation equations. The pressure sensor allowed direct measurement of pressure differential across the CV.

### Region of Interest Segmentation

The results of ROI segmentation in the phantom are shown in Figure 3. The method entailed examining intensity values in the magnitude image with the greatest contrast, Figure 3(a), to obtain a static (i.e. time invariant) ROI. Applying a threshold value of 0.60 results in a black and white image, Figure 3(b). By manually selecting the desired bright region the ROI is chosen, Figure 3(c), and contained *N *= 32 pixels with an area of 1.28 *cm*^2^, close to the cross-sectional area of the cylindrical passage . Comparison of perimeter pixels of the ROI to the original magnitude image, Figure 3(d), confirms the size, shape, and location of the segmented ROI match the location of the cylindrical passage.

### Mass Conservation

Mass conservation, Equation (1), enforces the observation that mass cannot be created or destroyed. For an incompressible fluid it may be used to relate volume flow rate to the time varying volume of fluid compartments and the net production or absorption within. Mass production and absorption are zero in the phantom. Therefore  in Equation (1), which assuming incompressibility (ie. *ρ *= *constant*), simplifies to a balance between the time-rate of change of volume and the volume flow rate periodically exiting and entering the CV. Volume flow rate was estimated from PC-MR velocity measurements in the phantom.

**Figure 4:**
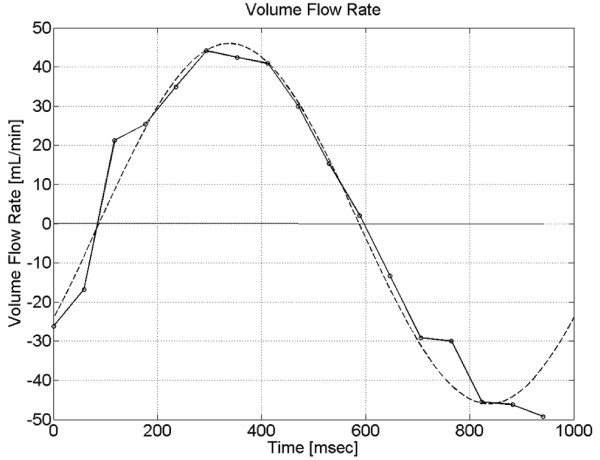
**Computed volume flow rate waveform**. Volume flow rate waveform, computed from PC-MR velocity data obtained in the phantom. *Q^k^*, in *mL*/*min*, was calculated using Equation (3) within the ROI shown in Figure 3(c) during a single cycle in *msec*. Flow into the phantom is positive. Dotted line displays sinusoidal fit.

Figure 4 shows the volume flow rate waveform, *Q^k^*, computed using Equation (3) within the ROI shown in Figure 3(c). Flow into the phantom is positive, as defined by the axial direction in Figure 2. The computed volume flow rate waveform agrees with sinusoidal behavior at a frequency of 1 *Hz*, shown as a dashed line in the figure. The normalized rms deviation between the measured waveform and sinusoidal fit was 6.3% or 5.8 *mL*/*min*. The actual Reynolds and Womersley numbers in this study were, *Re *= 77 and *α *= 15.9, respectively. The Reynolds number was less than design specification because of radial expansion of the tubing connecting the pump to the phantom, which decreased the amplitude of the flow waveform within the phantom.

Typical SNR for data sets used in this study were approximately 25. Using the relation for the velocity noise standard deviation [18,24], *σ_v _*= 0.36 *cm*/*s *when *V_enc _*= 20 *cm*/*s*. Therefore, the uncertainty in *Q^k ^*due to noise in the velocity measurement was .

### Momentum Conservation

Momentum conservation, Equation (2), was used to derive pressure differential from PC-MR velocity measurements. In addition, pressure differential was independently measured using a high resolution sensor for direct comparison with the CV approach.

**Figure 5:**
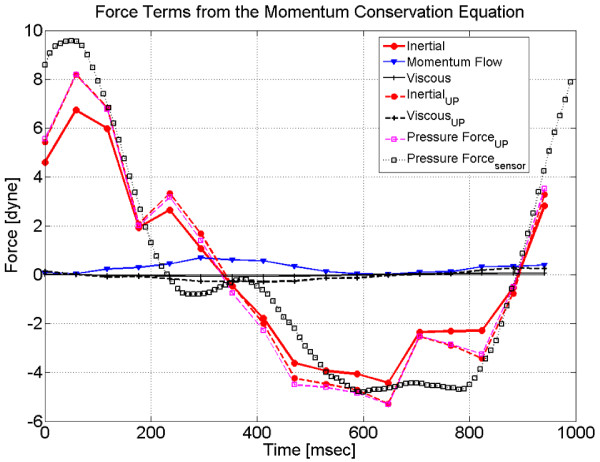
**Comparison of PC-MR derived pressure and sensor measurements**. Terms in the momentum conservation equation for the phantom control volume in *dyne *during a cycle in *msec*. Inertial, momentum flow (inflow only), and viscous force terms were estimated from axial PC-MR imaging in the phantom. Comparison to the method of Urchuk and Plewes (UP) [24] for the inertial, viscous, and pressure force are also shown. Good agreement between the CV and Urchuck/Plewes method for the computed inertial force and hence pressure force is observed. The measured pressure force was calculated directly from the pressure sensor output, Figure 6, and the flow area of the phantom.

Figure 5 displays the inertial, momentum flow (inflow only), and viscous force terms, computed from PC-MR data using Equations (4) - (6) in the phantom ROI, Figure 3(c). During experiments the phantom was static and the axial direction was horizontal, therefore gravity and acceleration, term (*iii*) in Equation (2), was zero in the axial momentum balance. In addition, the force due to fluid flow, term (*ii*) in Equation (2), is negligible in short conduit control volumes because the inflow and outflow of momentum balance in these cases. And for pulsatile flow at high Womersley number, by definition, the viscous force will be small in comparison to the inertial force [28]. As expected, the measured momentum (in)flow and viscous force waveforms, shown in Figure 5 confirm these terms are small compared to the inertial force. Therefore, momentum conservation may be simplified for pulsatile flow in conduit control volumes; the inertial force balances the pressure force. Uncertainty in the PC-MR derived inertial/pressure force estimate due to noise was .

**Figure 6:**
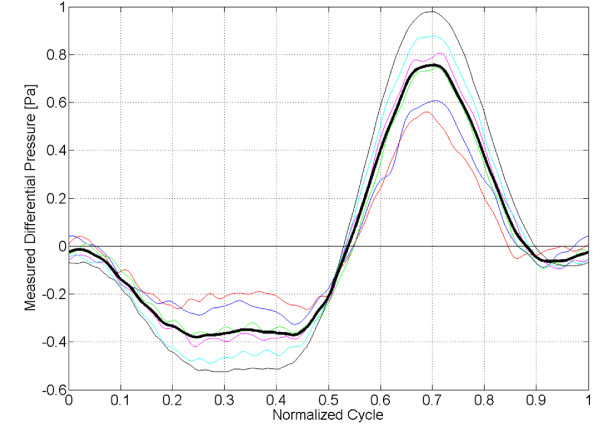
**Pressure differential sensor measurements**. Mean pressure differential waveform in *Pa *measured by sensor. Phase averaged pressure waveforms measured by the differential pressure sensor from six independent experiments are shown as thin lines. The ensemble average of these six experiments is shown as the thick back line.

Figure 6 displays differential pressure sensor measurements obtained across the CV in the phantom. Each thin line shows the phase average over 12 cycles of a single experiment. The thick black line displays their ensemble average, providing an estimate of the mean differential pressure waveform in the phantom, . The average uncertainty in the measured pressure differential due to noise and (phase and ensemble) averaging was *σ*_Δ*p *_= 0.10 *Pa*. The measured pressure force in Figure 5 was found as , the product of the mean pressure differential waveform, Figure 6 and the cross-sectional area of the CV. The corresponding uncertainty in the directly measured pressure force on the control volume was . The measured differential pressure force had amplitude of 14.4 *dynes *(pressure gradient amplitude 0.30 *Pa*/*cm*). Good agreement is observed between the PC-MR/CV derived (i.e. inertial) and directly measured pressure force in Figure 5. The normalized rms deviation between these waveforms was 12.5% or 1.8 *dynes *which is less than the sum of the uncertainties of these two waveforms. This serves as a proof-of-concept for the control volume framework which provides a method to non-invasively estimate pressure differential from PC-MR velocity measurements.

## Discussion

Control volume analysis is of great potential utility within the clinical community. Non-invasive estimation of pressure differential is merely one way CV analysis may be utilized. Depending upon the choice of CV, different terms in the momentum conservation equation are zero by definition or are shown to be negligibly smaller than other terms. Put another way, the physics occurring in the control volume dictate the terms remaining in the balance equations. Therefore, choosing a CV which will provide meaningful information, and upon which measurements are feasible, is a principle concern of the investigators.

For short conduit control volumes, such as the cylindrical passage in the phantom, the momentum inflow nominally cancels the outflow at the opposing CS, i.e. momentum flow contributes when there is a single in/outflow surface, large changes in velocity profile between entrance and exit, or extreme changes in the bulk flow direction occurs within the CV. In this case, the velocity profile on the inflow and outflow surfaces are expected to be similar and the overall momentum flow term, inflow minus outflow, will nominally be zero. Figure 5 shows the momentum flow term for one of these surfaces, not the difference between inflow and outflow of momentum, which would be much smaller and can therefore be neglected.

Previous investigations have noted that the contribution from the viscous force is small in comparison to the inertia of the oscillating fluid column [10,24]. Consistent with previous studies, the viscous force on the control volume was relatively small, as shown in Figure 5. This was expected at large Womersley number, *α *≳ 2.5, for which the transient inertial forces should dominate the viscous forces by definition [28,29].

Control volume analysis and the published method of Urchuk and Plewes [24] produced similar waveforms for the inertial and viscous contribution to the pressure force shown in Figure 5. While the methods used to compute these terms was quite different, the same conservation principles were being enforced. Using analysis provided by Urchuk and Plewes, the average uncertainty of the pressure estimate due to noise in the velocity images was [24]. This is nearly equal to the uncertainty of the control volume estimate, , above. The rms deviation of the Urchuk/Plewes pressure force estimate with respect to the measured pressure force was 11.2% or 1.6 *dyne*, compared to 8% reported in [24]. Similarly, the rms deviation between the control volume and Urchuk/Plewes inertial estimate was 4.8% or 0.7 *dyne *and between the CV and Urchuk/Plewes shear force estimates was 0.14 *dyne *or 25.6%. While Urchuk/Plewes and our CV method yielded very similar estimates, the utility of CV analysis is much greater than just this particular application; it was selected because it provided a direct comparison to established method.

Propagated uncertainty in the volume flow rate waveform included the effect of noise in the velocity measurement. As mentioned in the PC-MR post-processing section, there is also error introduced by using discretely sampled velocity data to evaluate terms in the conservation equations. This affect was studied independently by simulating velocity images in MATLAB using Womersley's exact solution [28] with similar parameter values found in the phantom experiments: *Re *= 77, *α *= 16, pixel size (0.2 *cm*), ROI area (1.48 *cm*^2^, 37 pixels), and flow amplitude (46 *mL*/*min*). The analytic solution was compared to the volume flow rate computed using Equation (3) throughout the image time series. The average error due to spatial resolution was 6.1% relative to the analytic solution at these conditions which is less than the uncertainty due to velocity noise. Discritization error will be a function of spatial and temporal resolution, and the Womersley number as discussed in [24]. At high Womersley number the velocity profile is relatively flat for a majority of the radial coordinate [28], fortunately allowing reliable volume flow estimates, however making the viscous shear force difficult to determine accurately because of the large velocity gradients near the wall. This results in decreased error in pressure estimation using CV analysis in short conduit control volumes with pulsatile flow because inertia is estimated from the measured volume flow waveform.

Pressure and flow have been related in previous models of CSF dynamics. Common to both bulk and pulsatile explanations of hydrocephalus, pressure differentials are required to drive CSF flow. Differences arise when considering the spatial and temporal scales and the fluid dynamic and physiologic parameters involved in the pressure differentials and fluid flow. The most common phenomenological models used hydraulic resistance to relate pressure drop to flow rate, Δ*P *= *QR *[12,30]. In using this relation bulk flow theorists implied that the pressure difference and flow waveforms were in phase at all times. When pulsatile dynamics are considered, complex variables were used to account for the phase difference between pressure and flow. The conservation equations impose a rigorous constraint on the pressures on the control volume, that they balance the sum of all other forces acting on the CV. In the phantom CV, the inertial force and pressure force balance and therefore the pressure and flow are out of phase.

## Conclusions

Diagnosis and treatment of hydrocephalus is hindered by a lack of systemic understanding of the interrelationships between pressures and flow of cerebrospinal fluid in the brain. Control volume analysis provides a fast, scientifically rigorous method for relating fluid flow and pressure information. Results of phantom experiments show agreement (12.5% normalized rms deviation) between control volume derived and directly measured pressure differential *in vitro *which serves as a proof-of-concept for determining *in vivo *pressure differentials non-invasively using control volume analysis and flow-sensitive magnetic resonance imaging.

## Abbreviations

CS: control surface; CSF: cerebrospinal fluid; CV: control volume; ICP: intracranial pressure; MR: magnetic resonance; PC-MR: phase-contrast magnetic resonance; rms: root mean square; ROI: region of interest; SNR: signal to noise ratio.

## Competing interests

The authors declare that they have no competing interests.

## Authors' contributions

BC designed and performed the phantom experiments, pressure measurements, data analysis, and drafted the manuscript. AV performed MR measurements for the phantom experiments. TW aided in pressure experiments, PC-MR processing, and drafting the manuscript. All authors have read and approved the final version of the manuscript.

## Supplementary Material

Additional file 1 **PC-MR post-processing methodology**. Mathematical formulation of PC-MR data processing methods. Click here for file
