# Supplementary material for: Magnetic resonance velocity imaging derived pressure differential using control volume analysis
Source: Fluids Barriers CNS. 2011 Mar 17;8:16. doi: 10.1186/2045-8118-8-16 (PMC3077315; doi:10.1186/2045-8118-8-16)
Supplement: Additional file 1 — PC-MR post-processing methodology. Mathematical formulation of PC-MR data processing methods. [file 2045-8118-8-16-S1.PDF]

Additional File: PC-MR post-processing methodology

Benjamin Cohen, Abram Voorhees, Timothy Wei

This supplementary file presents new methods to analyze PC-MR velocity data using the principles of control volume analysis. PC-MR phase and magnitude images were imported into MATLAB for image processing. Spatiotemporal velocity values were derived from the phase images as follows. A static background region was manually selected to determine the phase value corresponding to zero velocity, computed as the average phase value over space and time in this rectangular region. The remaining velocity values were linearly scaled using V_{enc} and the number of phase encoding steps of the data set. The standard deviation of phase values in this region provided an estimate of the velocity noise level, used to compute the signal to noise ratio (SNR). Custom software implemented in MATLAB was used to determine the region of interest and terms in the conservation equations as follows.

A region of interest (ROI) was segmented from the PC-MR data sets. Ideally the ROI contains only pixels within the lumen of the vessel of interest and represents the portion of the control surface through which fluid flows. The ROI may be assumed static or a function of time. Figure 3 outlines the process used for static ROI segmentation. The simplest method entails examining a magnitude image, Figure 3(a). A threshold value, between zero and one, was selected to create a binary (black and white) image, Figure 3(b), from which the ROI was manually selected, leaving only the desired ROI, Figure 3(c). Terms in the conservation equations were estimated from velocity values within the ROI as follows.

Of terms derived from PC-MR velocity measurements, the volume flow rate waveform is the simplest and most commonly computed. The product of image-normal or through-plane velocity, V_{ij}^k, and area, ΔS, for a pixel, (i, j), is the volume flow rate through that pixel at the kth acquisition time. Summation of pixels within the ROI yields the volume flow in a given pathway (e.g. aqueduct, spinal canal, blood vessels, or flow phantom) at the kth instant. Data throughout the cardiac cycle allows the volume flow rate waveform, $Q(t)$, to be estimated within the ROI for the k time intervals.

$$Q(t) = \int_{CS} \mathbf{u} \cdot \hat{\mathbf{n}} \, dS \simeq \sum_{(i,j)\in ROI} V_{ij}^k \Delta S = \overline{V}^k S = Q^k \tag{3}$$

For a static ROI of area, $S = N\Delta S$, the volume flow rate waveform may be written in terms of the mean velocity in the ROI, $\overline{V} = \frac{1}{N}\Sigma_{n=1}^{N} V_n$, as shown in Equation (3). The rms value of the mean velocity waveform was used as the signal magnitude when computing the SNR. In addition, the dot product in the integral term was simplified by

using through-plane velocities ($\mathbf{u} \cdot \hat{\mathbf{n}} \simeq \pm V_{ij}^k$), to be positive or negative for fluid exiting or entering a control volume, respectively.

Momentum flow, term (*ii*) in Equation (2), represents the force flowing fluid imparts on a control volume as it crosses the control surface. Computation of the axial momentum flow waveform is similar to volume flow, however the velocity values at each pixel were squared and multiplied by the fluid density.

$$\dot{P}_{axial}(t) = \int_{CS} \rho u \, (\mathbf{u} \cdot \hat{\mathbf{n}}) \, dS \simeq \sum_{(i,j) \in ROI} \rho (V_{ij}^k)^2 \Delta S = \dot{P}^k \tag{4}$$

Here ROI represents the same region used in the volume flow calculation, Equation (3). The nominal density of water, $\rho_o = 1 \; g/cm^3$, was used for phantom experiments.

The time-rate of change of momentum within a CV, term (*i*) in Equation (2), may vary for three reasons: due to temporal variations of 1) the fluid velocity field, 2) the fluid density and 3) the CV spatial extent. For pulsatile flow of an incompressible fluid in tube-like control volumes considered in this study, as a first approximation, the former contribution will significantly exceed the latter two. The axial momentum equation was used to determine the inertial force on the CV from the computed volume flow rate waveform as follows.

$$I_{axial}(t) = \frac{\partial}{\partial t} \int_{CV} \rho u \, dV \simeq \rho L \frac{dQ^k}{dt} = I^k \tag{5}$$

Here $dV = LdS$ and L is the axial length of the CV shown in Figure 2. It is assumed that no axial variation in the velocity profile exists, allowing the inertial force to be estimated from axial PC-MR data in a single slice through the CV. The temporal derivative was evaluated by central differencing of the discrete volume flow rate waveform, Q^k, computed using Equation (3).

The viscous shear force, term (*v*) in Equation (2), represents drag on the control surface due to fluid friction. To compute the shear force on the CV the shear stress at the wall, $\boldsymbol{\tau}_w = \mu \frac{\partial \mathbf{u}}{\partial n}\Big|_w = \mu \nabla \mathbf{u} \cdot \hat{\mathbf{n}}_w$, must be integrated over the control surface. The shear stress was estimated from the gradient of the through-plane velocity projected onto the inward surface normal vector, $-\hat{\mathbf{n}}$, of the ROI. This computation is equivalent to the linear extrapolation method commonly used for the ordinate directions; however the technique was generalized to allow computation of the wall normal velocity gradient in any direction. Summation of the product of shear stress and pixel edge length, Δs, within the perimeter pixels of the ROI and multiplication by L provides an estimate of the axial shear force on the CS.

$$S_{axial}(t) = \int_{CS} \boldsymbol{\tau}_w dS \simeq \mu L \sum_{(i,j)\in P} (\nabla_s V_{ij}^k \cdot -\hat{\mathbf{n}}_{ij})\Delta s = S^k \qquad (6)$$

Here P are the perimeter pixels of the ROI, shown in Figure 3(d), $dS = Lds$ and ∇_s is the discrete, central difference spatial gradient operator. Viscosity was assumed to be nominally that of water, $\mu_o = 0.01\ g/cm\ s$, in the phantom.

Approximate equality between integral expressions and discrete summations (i.e. $\simeq$) in Equations (3)-(6) reflects errors which are naturally introduced by spatial and temporal discretization of the velocity field. The method of Urchuk and Plewes, for estimating pressure gradient waveforms from PC-MR data, was used to compare to terms estimated by Equations (3)-(6). Finite difference operations were executed in MATLAB. The spatial averaging region was computed as the ROI minus the perimeter pixels, representing the internal portion of the lumen. For direct comparison with CV waveforms the pressure gradient waveform, and inertial and viscous contributions, were multiplied by SL, the volume of the CV.
